# Caffeine is associated with improved alveolarization and angiogenesis in male mice following hyperoxia induced lung injury

**DOI:** 10.1186/s12890-019-0903-x

**Published:** 2019-07-30

**Authors:** Vikramaditya Dumpa, Lori Nielsen, Huamei Wang, Vasantha H. S. Kumar

**Affiliations:** 1 Winthrop University Hospital, Mineola, NY USA; 2 Division of Neonatology, Department of Pediatrics, John R Oishei Children’s Hospital, University at Buffalo, 1001 5th Floor Main Street, Buffalo, NY 14203 USA

**Keywords:** Caffeine, Newborn, Mice, VEGF, Hypoxia-inducible factors, Lung, Hyperoxia

## Abstract

**Background:**

Caffeine therapy for apnea of prematurity reduces the incidence of bronchopulmonary dysplasia (BPD) in premature neonates. Several mechanisms, including improvement in pulmonary mechanics underly beneficial effects of caffeine in BPD. As vascular development promotes alveologenesis, we hypothesized that caffeine might enhance angiogenesis in the lung, promoting lung growth, thereby attenuating BPD.

**Methods:**

C57Bl/6 mice litters were randomized within 12 h of birth to room air (RA) or 95%O_2_ to receive caffeine (20 mg/kg/day) or placebo for 4 days and recovered in RA for 12wks. The lung mRNA and protein expression for hypoxia-inducible factors (HIF) and angiogenic genes performed on day 5. Lung morphometry and vascular remodeling assessed on inflation fixed lungs at 12wks.

**Results:**

Caffeine and hyperoxia in itself upregulate HIF-2α and vascular endothelial growth factor gene expression. Protein expression of HIF-2α and VEGFR1 were higher in hyperoxia/caffeine and angiopoietin-1 lower in hyperoxia. An increase in radial alveolar count, secondary septal count, and septal length with a decrease in mean linear intercept indicate an amelioration of hyperoxic lung injury by caffeine. An increase in vessel surface area and a significant reduction in smooth muscle thickness of the pulmonary arterioles may suggest a beneficial effect of caffeine on vascular remodeling in hyperoxia, especially in male mice.

**Conclusions:**

Postnatal caffeine by modulating angiogenic gene expression early in lung development may restore the pulmonary microvasculature and alveolarization in adult lung.

## Background

Bronchopulmonary dysplasia (BPD), a form of chronic lung disease is a common cause of morbidity and mortality in extremely premature infants. Multiple factors are implicated in the pathogenesis of BPD including chorioamnionitis, oxygen toxicity, mechanical ventilation, and sepsis [1]. Irrespective of the pathogenesis, impaired alveolarization, and dysregulated angiogenesis are the defining characteristics of BPD [2]. Increased risk for asthma, related respiratory hospitalizations, and poor neuro-developmental outcomes are some of the long-term morbidities associated with BPD [3, 4].

Caffeine therapy in very low birth weight (VLBW) infants decreases BPD [5] and improves survival without neurodevelopmental disability at 18-month follow-up in these infants [6]. However, caffeine had no effect on the primary outcome of death or survival with a severe disability when the children were 5 years of age [7]. Caffeine treatment in premature infants not only reduces apnea of prematurity and recurrent hypoxic episodes in infants [8] but also reduces lung injury and improves respiratory function in children [9].

Caffeine is a nonspecific adenosine receptor antagonist. Adenosine is a purine nucleoside-signaling molecule with effects on regulating fibrosis, angiogenesis, and inflammation. Caffeine is thought to modulate TGF-β signaling [10], decrease inflammation [11] or attenuate endoplasmic stress [12] in protecting the lung from hyperoxia-induced lung injury [12, 13]. However, several studies have suggested an adverse role for caffeine in lung development [10, 14]. Although the molecular mechanisms underlying prevention of BPD with caffeine are uncertain, the potential beneficial effects could include antagonist effects of the adenosine receptor subtypes, A_1_, A_2a_, A_2B,_ and A_3_ [15]. Adenosine promotes angiogenesis and regulates the expression of vascular endothelial growth factor (VEGF) through the adenosine receptors in different cell types [16, 17] and the regulation of hypoxia-inducible factor (HIF) pathway [16, 18, 19]. Recent studies suggest that disruption of VEGF function plays a pivotal role in the pathogenesis of BPD [20, 21]. Hypoxia-inducible factor-1 regulates the expression of genes encoding vascular development, with VEGF and angiopoietins being the important ones [22, 23]. HIF-1 expression is tightly linked to the oxygen concentration in vivo and hyperoxia or even normoxia in the developing lung rapidly induce HIF degradation and hence, VEGF expression [24]. The effects of caffeine on the developing lung vasculature in premature infants are not determined. A caffeine loading dose of 20 mg/kg would be approximately equivalent to 14–16 cups of coffee, and the maintenance dose (5 – 10 mg/kg) equivalent to 4–8 cups of coffee in an adult. Given these high doses over prolonged periods, our objective was to study whether caffeine modulates the HIF-1α/angiogenesis pathway contributing to an improvement in lung alveolarization. Lungs of adult mice exposed to hyperoxia as newborns are simplified and exhibit reduced function much like that observed in children with BPD as infants [25]. We investigate the effects of caffeine administration on angiogenesis and lung morphometry in newborn mice exposed to hyperoxia, a lung injury model similar to BPD in premature infants.

### Methods

The Institution Animal Care and Use Committee of the University at Buffalo (Project # PED24116N- Current) approved all experimental protocols before the study. Time**-**dated pregnant C57Bl/6 mice were acclimatized in the lab animal facility after purchasing from the vendor (Envigo RMS Inc., Indianapolis, IN) before delivery. Dams were observed on the day of expected delivery for delivery of new litters with minimal disturbance. To minimize litter-dependent bias, all pups from different litters were distributed randomly between dams (5–6 pups/dam) within 12 h of birth into four groups: hyperoxia-caffeine (*HC*), room air-caffeine (*RAC*), hyperoxia-saline (*HS*) and room air-saline (*RAS*) groups (3–4 litters/group; Minimum of 18 pups/group). Litters were exposed to hyperoxia (95% O_2_) for 96 h from Day 1 to Day 4 (*HS/HC*) or to room air (*RAS/RAC*) to serve as controls. Oxygen exposures were performed in a large Plexiglas chamber monitored for temperature, oxygen concentration (95%O_2_ or 21%O_2_), and humidity (50–60%). We alternated dams between hyperoxia and room air-exposed litters every 24 h to prevent maternal oxygen toxicity. Mice in the room air group were subjected to the same environment as the hyperoxia group. All pups were weighed at birth and every day after that. Caffeine citrate (20 mg/mL; Fresenius Kabi, Lake Zurich, IL) was administered at a dose of 20 mg/kg/day for mice randomized to the caffeine groups (*RAC/HC* groups) or an equivalent volume of normal saline for mice randomized to saline (*RAS/HS* groups) by intraperitoneal injection. All doses were administered at the same time (10 AM to Noon) once every 24 h for four doses. Mice were sacrificed 24 h after the last treatment of caffeine on postnatal day 5 with an intraperitoneal injection of sodium pentobarbital. Gene expression, protein, and cytokine analysis were performed in mice sacrificed on Day 5 in all the four groups (eight mice/group). In the second part of the experiment, mice recovered in room air following 96 h of hyperoxia and postnatal caffeine for 12 weeks until sacrifice. Lungs were formalin fixed at 12 weeks in all groups (ten mice/group).

### RNA isolation

RNA was isolated from flash frozen mouse lung using RNeasy mini kit (Qiagen, Valencia, CA) with on-column DNase digestion per manufacturer’s protocol. RNA integrity was assessed using the Experion Automated Electrophoresis System (Biorad, Hercules, CA).

### Real-time quantitative PCR

Genes of the hypoxia-inducible factor (HIF) pathway (HIF-1α, HIF-2α, HIF-3α, HIF-1β & prolyl hydroxylase-2) and selected genes of the angiogenic pathway (VEGF: vascular endothelial growth factor; VEGFRR1/fLT-1: VEGF receptor; ANG1: angiopoietin-1) were analyzed by real-time quantitative PCR. Total cellular RNA was reverse transcribed using the iScript cDNA synthesis kit (Biorad, Hercules, CA). Reactions without reverse transcriptase were included for individual RNAs as negative controls. Primers for the above genes were purchased from Real Time Primers (Elkins Park, PA) and the most stable reference genes phosphoglycerate kinase 1 and peptidylprolyl isomerase A were chosen from a panel of ten genes using geNorm software (Biogazelle, Belgium). Reactions were run in duplicate in a CFX Connect Real-Time PCR detection system (Biorad Lab Inc.) using SYBR-Green to measure DNA amplification. The instrument’s software was used to calculate the threshold cycle (C_t_) values for all the genes on the PCR Array. Fold change in gene expression for pair-wise comparison was processed on the Excel-based PCR Array Data Analysis software (SA Biosciences, MD) using the eq. 2^-∆∆Ct^ by comparing the control group (*RAS*) to the other three groups (*RAC, HS, HC*).

### Angiogenic protein analysis

Snap-frozen lung tissue was homogenized in ice-cold PBS (pH – 7.4) with protease inhibitors (Sigma, St. Louis, MO), spun, and the supernatant used for protein analysis by ELISA. The protein concentration was determined using the DC protein assay (BioRad, Hercules, CA). Protein analysis for HIF1α, HIF2α and HIF3α proteins (NovaTein Biosciences, Woburn, MA) and VEGF, VEGFR1 and ANG1 protein (R&D Systems, Minneapolis, MN) were performed by enzyme-linked immunosorbent assay (ELISA) according to manufacturer’s protocol.

### Cytokine measurements

Tumor necrosis factor α (TNF- α), Interferon γ (INF-γ) & interleukin-6 (IL-6) were measured in whole lung homogenates from cell-free supernatants on a postnatal day 5 in all the groups. Cytokine levels performed by ELISA technique (R&D systems, Minneapolis, MN), were normalized to total lung protein measured by Lowry assay.

### Lung histopathology

The lungs were instilled with 10% buffered formalin at 25 cm H_2_O pressure in adult mice following tracheal cannulation. Instilled lungs were fixed overnight, serially dehydrated in ethanol and embedded in paraffin. Lung tissue cut into 5 μm full sections and stained with hematoxylin & eosin. We analyzed five slides/mouse and randomly analyzed 20 fields/slide for lung morphometry in all the groups. Alveolar development was evaluated by assessment of radial alveolar count (RAC) [26] and mean linear intercept (MLI) [27] by Aperio image analysis software (Leica Biosystems Inc., Buffalo Grove, IL). Assessment of secondary septal crest density performed by counting of secondary septae longer than 5 μm in high-resolution images (× 400, 57,600μm^2^); 20 HPF images assessed per slide and averaged for each animal to calculate the mean for each group [28, 29]. Similarly, elastin stained lung sections were evaluated for mean septal length in high-resolution images (x400x); the septal length was assessed from the base to the tip of the septum; 20 HPF/slide) by Aperio imaging software (Leica Biosystems Inc., Buffalo Grove, IL) [28].

### Assessment of lung vessels

Lung vasculature was assessed on lung sections stained for collagen (trichrome staining) and endothelial cells (Von Willebrand factor immune histo-chemistry) at 12wks. Vessels that accompanied the airways were characterized as pulmonary arteries. Pulmonary arterioles of 20 - 100 μm in diameter were included and bronchioles excluded for analysis by a single operator blinded to the study groups. The number of blood vessels/high power field (× 200; 237,600μm^2^) were counted successively in 20 HPFs per slide; the total blood vessel area (μm^2^) per HPF was also assessed in the same fields (200x) by Aperio image analysis software (Leica Biosystems Inc., IL, USA). Five lung sections were studied for each animal (*n* = ten pups per group).

### Smooth muscle actin (α-SMA) immunohistochemistry

We first performed antigen retrieval of lung sections with citrate solution followed by blocking of non-specific binding with 10% goat serum in phosphate buffered saline (PBS). Lung sections incubated overnight with antibody to α-SMA (mouse monoclonal antibody, 1:100 dilution; Sigma-Aldrich Co. St Louis, MO) at 4 °C and rinsed with PBS. The sections were then incubated with peroxidase-labeled rabbit anti-mouse IgG (1:1000) for 30 min at 37 °C, washed with PBS and stained with 3,3′-diaminobenzidine (Sigma-Aldrich Co). Assessment of wall thickness was performed on α-SMA stained pulmonary vessels of 20 - 100 μm diameter at 400x magnification in all the four groups. We used Aperio image analysis software (Leica Biosystems Inc., Buffalo Grove, IL) to measure the cross-sectional area of the pulmonary arteriole. The surface area of the vessel wall assessed as a fraction of the total vessel area by the following formula, Area _OVW_ – Area _IVW_/Area _OVW_; wherein, OVW – outer vessel wall, representing surface area of the cross-sectioned vessel; IVW – inner vessel wall, serving the surface area of the vessel lumen) [30].

### Statistical analysis

Replicated 2-∆∆^C(t)^ values for each gene in the control group and the treatment group was used to calculate *p* values by student’s t-test. More than two group comparisons performed by analysis of variance (ANOVA) with Fisher’s post-hoc analysis when appropriate (Stat view, Abacus Concepts, Piscataway, NJ). Repeated measures ANOVA used to assess differences in body weight over time among groups. All values expressed as mean ± standard deviation (SD) of the mean with *n* representing the number of animals studied. A *p* value of < 0.05 was considered significant.

## Results

### Growth

**Fig. 1:**
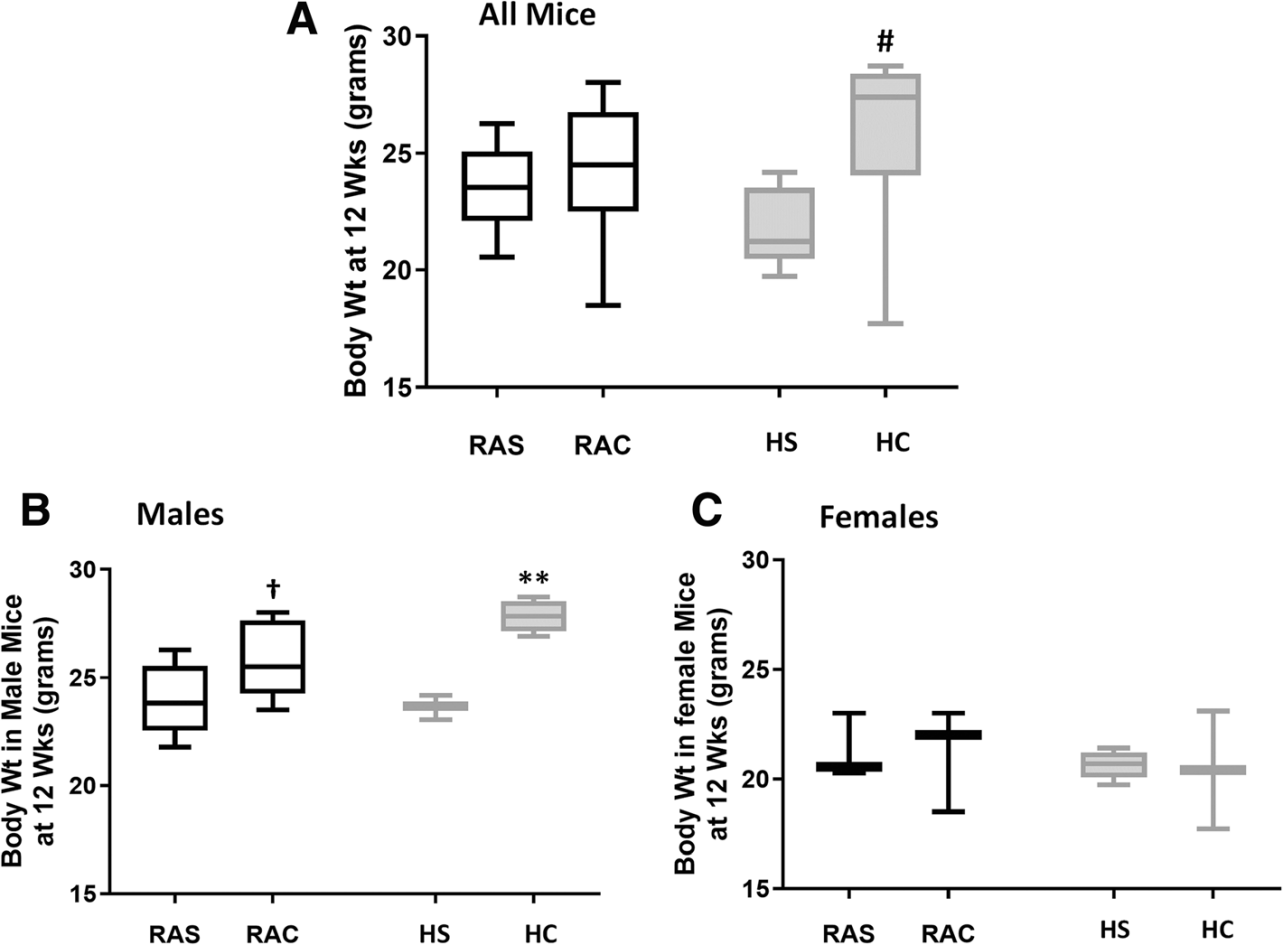
Body weights (grams) in adult mice at sacrifice in all the four groups (*Open box plots – Room air; shaded box plots – hyperoxia; RAS – room air saline; RAC – room air caffeine; HS – hyperoxia saline; HC – hyperoxia caffeine).* Caffeine treated mice had significantly higher body weight at 12wks in hyperoxia (#*p* < 0.01 vs. *HS* group, **a**). Hyperoxic male mice treated with caffeine had significantly higher body weight compared to other groups (**p < 0.001 vs. *RAS, RAC & HS* groups, **b**). Caffeinated male mice had significantly higher body weight compared to RA or hyperoxia controls (†*p* < 0.05 vs. *RAS* & *HS* groups). No significant difference in body weight noted in female mice (**c**). # ** † Fisher’s post-hoc test, ANOVA (*n* = ten mice per group) (*RAS* – 6 males, 5 females; *RAC* – 6 males, 4 females; *HS* – 5 males, 5 females; *HC* – 6 males, 4 females)

There was no difference in body weight at 12wks between the two room air groups (*RAS:* 23.52 ± 1.86 g vs. *RAC:* 24.22 ± 2.92 g; Fig. 1a). However, postnatal caffeine treatment significantly increased body weight in mice exposed to hyperoxia (*HS:* 21.77 ± 1.64 g vs. *HC:* 25.99 ± 3.77 g, *p* < 0.05 vs. *HS* group, ANOVA; Fig. 1a). Male mice gained significant body weight following postnatal caffeine administration. Mice exposed to room air (RA) and treated with caffeine had significantly higher body weight compared to both the saline groups (*RAC:* 25.79 ± 1.ra; *RAS:* 23.89 ± 1.64 g & *HS:* 23.64 ± 0.56 g; *p* < 0.05 vs. *RAS* & *HS* groups, ANOVA; Fig. 1b). However, in mice exposed to hyperoxia, postnatal caffeine significantly increased body weight compared to all the other three groups (*RAS:* 23.89 ± 1.64 g; *RAC:* 25.79 ± 1.75 g; *HS:* 23.64 ± 0.56 g & *HC:* 27.82 ± 0.82 g, *p* < 0.01 vs. *RAS, RAC* & *HS* groups, ANOVA; Fig. 1b). There was no significant difference in body weight at 12wks in female mice with or without caffeine (Fig. 1c). We studied ten mice per group (*RAS* – 6 males, 5 females; *RAC* – 6 males, 4 females; *HS* – 5 males, 5 females; *HC* – 6 males, 4 females).

### Gene expression

Gene expression was measured as fold change (FC) in mRNA expression relative to the control group (*RAS* group, FC of *RAS* group = 1, Table 1). Genes involved in the angiogenic pathway such as HIF-2α, PHD-2, and VEGF were significantly over-expressed in the room air caffeine and hyperoxia-saline groups compared to hyperoxia-caffeine group (Table 1). Expression of ANG-1 was considerably higher in room air mice exposed to caffeine (*RAC* group) compared to hyperoxia groups (Table 1). We observed no differences in the expression of HIF-1α, HIF-3α, HIF-1β or Flt-1 (VRGFR1) genes among the three groups.

**Table 1:** Expression of selected angiogenic genes in the lung by qRT-PCR in newborn mice exposed to hyperoxia (95%O_2_) or room air and administered caffeine or saline

Gene Symbol	Room Air-Caffeine (RAC) Group	Hyperoxia-Saline (HS) Group	Hyperoxia-Caffeine (HC) Group
Hif-1α	2.28 ± 1.05	2.31 ± 0.33	2.35 ± 0.85
Hif-2α	2.62 ± 0.87*	2.67 ± 0.44*	1.52 ± 0.70
Hif-3α	2.83 ± 1.25	1.72 ± 0.44	1.64 ± 0.71
Hif-1β	1.77 ± 0.34	1.65 ± 0.20	1.51 ± 0.43
PHD-2	1.78 ± 0.30*	1.89 ± 0.46*	1.00 ± 0.26
VEGF	2.00 ± 0.42*	3.52 ± 0.66*	1.17 ± 0.34
Flt-1	2.71 ± 0.89	2.44 ± 0.40	1.93 ± 0.57
Ang-1	2.85 ± 1.26*	1.89 ± 1.11	0.89 ± 0.27

The fold-change (FC) in gene expression is compared to the room air-saline (RAS) group (control group; FC in RAS = 1.0. (*n* = 8 pups in each group)

Values expressed as fold change ± SD compared to room air saline group. *p < 0.05 versus hyperoxia-caffeine group (Fisher’s post-hoc test, ANOVA); *Hif-1α* - Hypoxia-Inducible Factor-1α; *HIF-2α* - Hypoxia-Inducible Factor-2α; *HIF-3α* - Hypoxia-Inducible Factor-3α; *Hif-1β* - Hypoxia Inducible Factor-1β; *PHD-2*: Prolyl Hydroxylase – 2 (EGLN1); *VEGF* - Vascular Endothelial Growth Factor; *Flt-1*: VEGF Receptor 1; *Ang-1*: Angiopoietin-1. *Housekeeping genes* – Phosphoglycerate kinase-1 (*Pgk1*); Peptidylprolyl isomerase A (*Ppia*)

### Angiogenic proteins

Protein expression in the lung for HIF-1α, HIF-2α, HIF-3α, VEGF, FLT-1, and ANG1 was analyzed by enzyme immunoassay in 5-day old mice in all the four groups (Table 2). HIF-2α protein expression was significantly higher in the hyperoxia-caffeine group (*p* < 0.05 vs. *RAS & RAC* groups, Fisher’s post-hoc test, ANOVA; Table 2). Expression of VEGF protein was significantly higher in the hyperoxia group compared to the other three groups (*p* < 0.05 vs. *RAS, RAC & HC* groups, Fisher’s post-hoc test, ANOVA; Table 2). Expression of angiogenic receptor, VEGFR1 (FLT-1) protein was significantly higher in the hyperoxia-caffeine group compared to both the room air groups (*p* < 0.05 vs. *RAS & RAC* groups, Fisher’s post-hoc test, ANOVA; Table 2). Expression of ANG-1 protein was significantly lower in the hyperoxia group compared to the other three groups (*p* < 0.05 vs. *RAS, RAC & HC* groups, Fisher’s post-hoc test, ANOVA; Table 2). There were no differences in protein expression of cytokines, such as TNF-α, INF-γ, and IL-6 in the lung among the three groups (Table 2).

**Table 2:** Protein expression of selected angiogenic proteins and cytokines in the lung measured by enzyme immunoassay (EIA) in newborn mice exposed to hyperoxia (95%O_2_) or room air and administered caffeine or saline

Protein	Room Air Saline Group (RAS)	Room Air Caffeine Group (RAC)	Hyperoxia Saline Group (HS)	Hyperoxia Caffeine Group (HC)
HIF & Angiogenic Proteins (ng or pg/mg protein)				
Hif-1α (pg)	230 ± 25	215 ± 41	250 ± 40	253 ± 46
Hif-2α (ng)	0.232 ± 0.058	0.117 ± 0.008	0.274 ± 0.055	0.464 ± 0.159*
Hif-3α (pg)	6.96 ± 1.78	5.99 ± 0.51	7.60 ± 1.31	6.05 ± 0.78
VEGF (pg)	55.5 ± 16.1	64.6 ± 7.1	177.5 ± 52.8**	92.6 ± 16.4
Flt-1 (pg)	2297 ± 1026	1714 ± 85	3364 ± 1590	5866 ± 3086*
Ang-1 (ng)	522 ± 192	664 ± 72	258 ± 70†	455 ± 70
Cytokines				
TNF-α (pg/μg protein)	0.350 ± 0.01	0.040 ± 0.01	0.043 ± 0.02	0.045 ± 0.01
INF-γ (pg/μg protein)	0.098 ± 0.04	0.109 ± 0.03	0.118 ± 0.04	0.134 ± 0.02
IL-6 (pg/μg protein)	0.065 ± 0.02	0.062 ± 0.01	0.081 ± 0.04	0.066 ± 0.01

HIF & angiogenic proteins are expressed in ng or pg/mg protein of the lung (*n* = 8 pups in each group)

Values expressed as mean ± SD. * *p* < 0.05 vs RAC & RAS groups, **p < 0.05 vs RAS, RAC & HC groups, †*p* < 0.05 vs RAS, RAC & HC groups; (Fisher’s post-hoc test, ANOVA). *HIF-1α* - Hypoxia-Inducible Factor-1α; *HIF-2α* - Hypoxia-Inducible Factor-2α; *HIF-3α* - Hypoxia-Inducible Factor-3α; *VEGF* - Vascular Endothelial Growth Factor; *Flt-1*: VEGF Receptor 1*; Ang-1*: Angiopoietin-1; *TNF-α*: tumor necrosis factor alpha; *INF-γ*: interferon gamma; *IL-6*: interleukin-6. HIF & angiogenic proteins are expressed as units/mg lung protein

### Histopathology

**Fig. 2:**
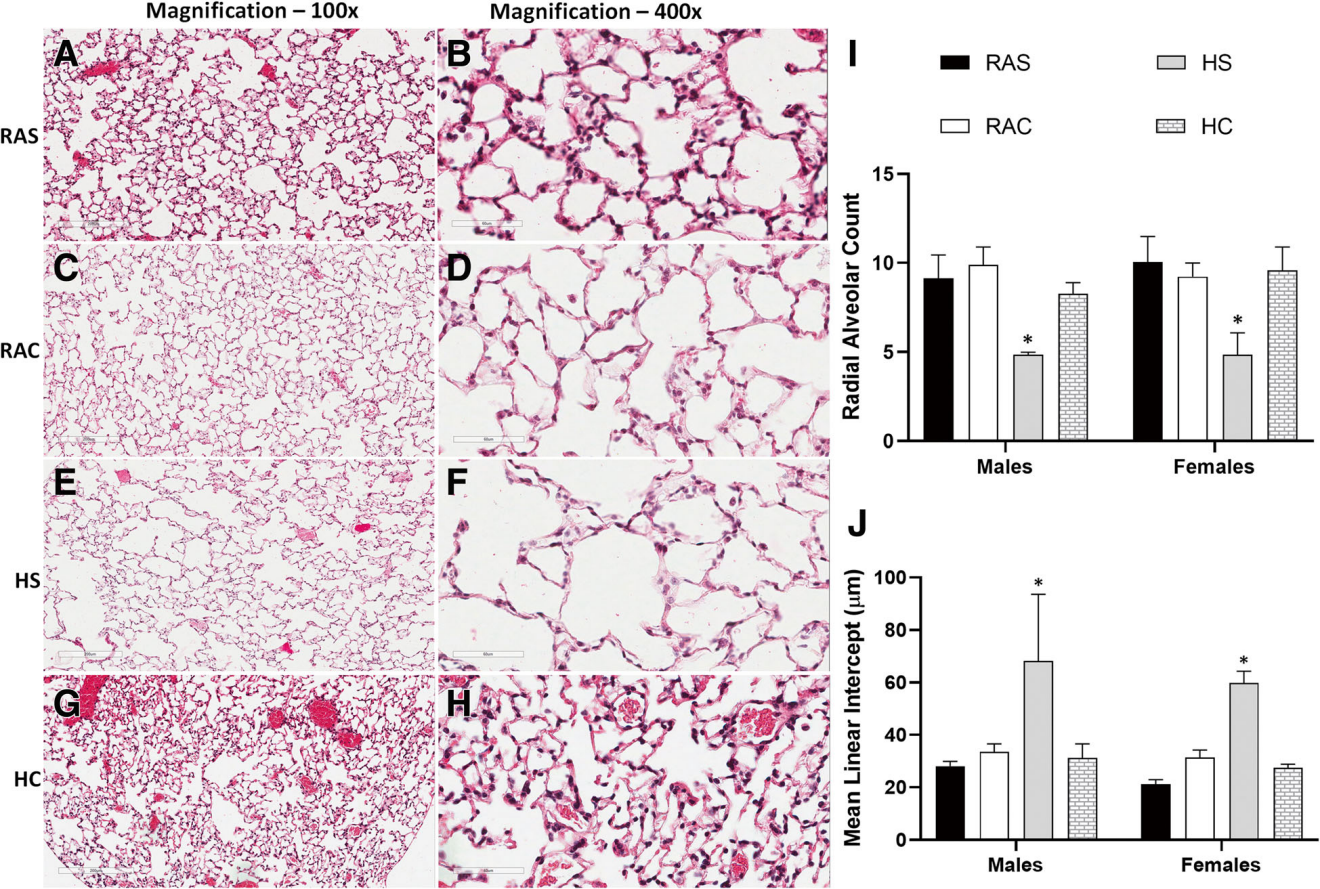
Histopathology of lungs by H&E at 12wks (100x - **a**,**c**,**e**,**g**; 400x - **b**,**d**,**f**,**h** in all the four groups (*room air saline (RAS)* – **a**/**b**; *room air caffeine (RAC)* – **c**/**d**; *hyperoxia saline (HS)* – **e**/**f**; *hyperoxia caffeine (HC)* – **g**/**h**). Alveoli were numerous in both the RA groups (**a**/**b**; **c**/**d**); hyperoxia impaired alveolar development with fewer, distended alveoli (E/F) and caffeine restored lung alveolarization (**g**/**h**; **i**/**j**) (*Scale bar*: 200 μm (100x), 60 μm (400x)). The radial alveolar count was significantly lower (**i**) and mean linear intercept was significantly higher (**j**) in the *HS* group in both male and female mice suggesting alveolar simplification (*Open box plots – RA groups; shaded box plots – hyperoxia groups*). *p < 0.001 vs. *RAS, RAC & HC* groups; Fisher’s post-hoc test, ANOVA (*n* = ten mice/group)

**Fig. 3:**
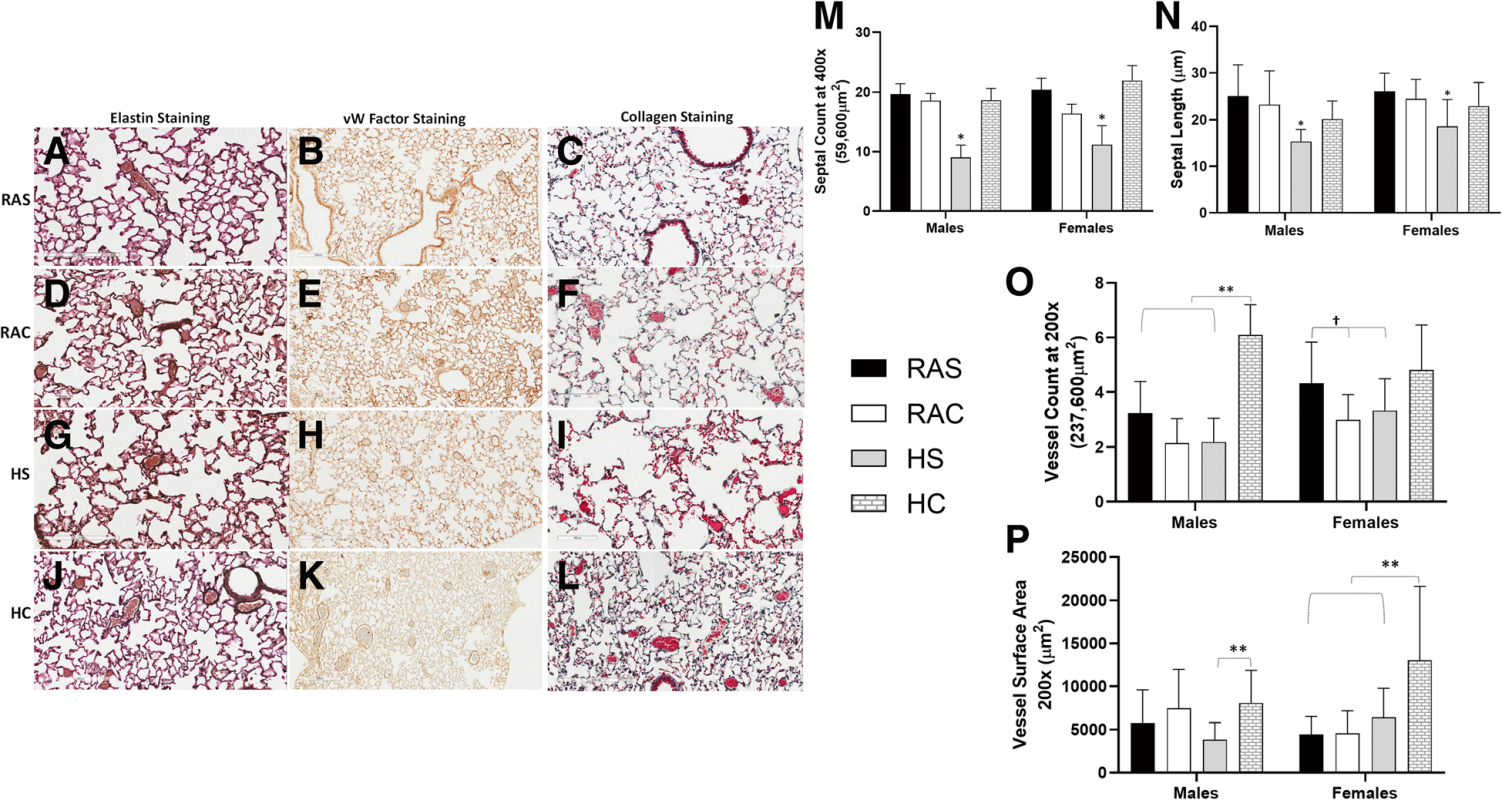
Representative sections of elastin (*left panel* – **a**/**d**/**g**/**j**), Von Willebrand factor (vWF) (*Middle panel* – **b**/**e**/**h**/**k**) and trichrome (*right panel* – **c**/**f**/**i**/**l**) staining of lung sections (200x) in all the four groups (*room air saline (RAS)* – **a**/**b**/**c**; *room air caffeine (RAC)* – **d**/**e**/**f**; *hyperoxia saline (HS)* – **g**/**h**/**i** & *hyperoxia caffeine (HC)* – **j**/**k**/**l**). Septal count (**m**) and septal length (**n**) were studied in elastin sections; vessel count (**o**) and vessel surface area (**p**) were evaluated by vW Factor immunohistochemistry and trichrome sections (*Open box plots – RA groups; shaded box plots – hyperoxia groups*). Lower septal count (**m**) and reduced septal length (**n**) in the hyperoxia group was significantly augmented by caffeine in both male and female mice (*p < 0.001 vs. *RAS, RAC & HC* groups, Fisher’s post-hoc test, ANOVA). Vessel count was significantly higher in the *HC* group, especially in male mice (o, **p < 0.0001 vs. *RAS, RAC & HS* groups, †*p* < 0.01 vs. *RAC* & *HS* groups; Fisher’s post-hoc test, ANOVA). Blood vessel surface area at 200x (237,600μm^2^) was higher in the hyperoxia group administered caffeine in both male and female mice (**p**, **p < 0.0001 vs *RAS, RAC & HS* groups, Fisher’s post-hoc test, ANOVA (*n* = ten mice/group)

Mice exposed to postnatal hyperoxia demonstrate distended alveoli and enlarged air sacs at 12wks of age (Fig. 2e/f) when compared to relatively well-formed alveolar units in the RA groups (Fig. 2: a–d). Radial alveolar count (RAC), a unit of alveolar development was significantly lower in mice exposed to hyperoxia suggesting alveolar simplification (*p < 0.001 vs. *RAS & RAC* groups; Fisher’s post-hoc test, ANOVA; Fig. 2i). Mean linear intercept (MLI), a unit of alveolar enlargement was significantly higher in the hyperoxia group (*p < 0.001 vs. *RAS & RAC* groups, Fig. 2j). Postnatal caffeine augmented postnatal alveolarization and attenuated alveolar distension following hyperoxia (*p < 0.001 vs. *HC* group, ANOVA; Fig. 2i), suggesting caffeine may ameliorate hyperoxic lung injury in adult mice (Fig. 2g/h). A significant increase in secondary septal count (*p < 0.001 vs. *HC* group, Fisher’s post-hoc test, ANOVA; Fig. 3m) and septal length (*p < 0.001 vs. *HC* group; Fig. 3n) further strengthens the protective effects of postnatal caffeine administration. We demonstrate the beneficial effects of caffeine on all markers of alveolar development in both male and female mice (Fig. 2/Fig. 3). The findings advance the protective effects of caffeine against hyperoxia-induced lung injury and promoting lung growth.

### Vessel data

Pulmonary blood vessels of 20-100 μm diameter were counted following von Willebrand factor immunohistochemistry and in trichrome stained lung sections in all the groups (Fig. 3). Blood vessel density expressed as a number of blood vessels per field studied was significantly higher in the hyperoxia group following postnatal caffeine, especially in male mice (**p < 0.0001 vs. *RAS, RAC and HS* groups, Fisher’s post-hoc test, ANOVA; Fig. 3o). Control female mice had significantly higher vessel density compared to *RAC* and *HS* groups (†*p* < 0.001 vs. *RAC and HS* groups, Fisher’s post-hoc test, ANOVA; Fig. 3o); however, not different from hyperoxic mice that received postnatal caffeine. The surface area of blood vessels (237,600μm^2^) was significantly higher in both male and female mice that received postnatal caffeine and hyperoxia (**p < 0.0001 vs. *RAS, RAC and HS* groups, Fisher’s post-hoc test, Fig. 3q). The finding suggests that postnatal caffeine promotes microvascular growth along with alveolar growth.

### Caffeine & Pulmonary Arterial Wall Thickness

**Fig. 4:**
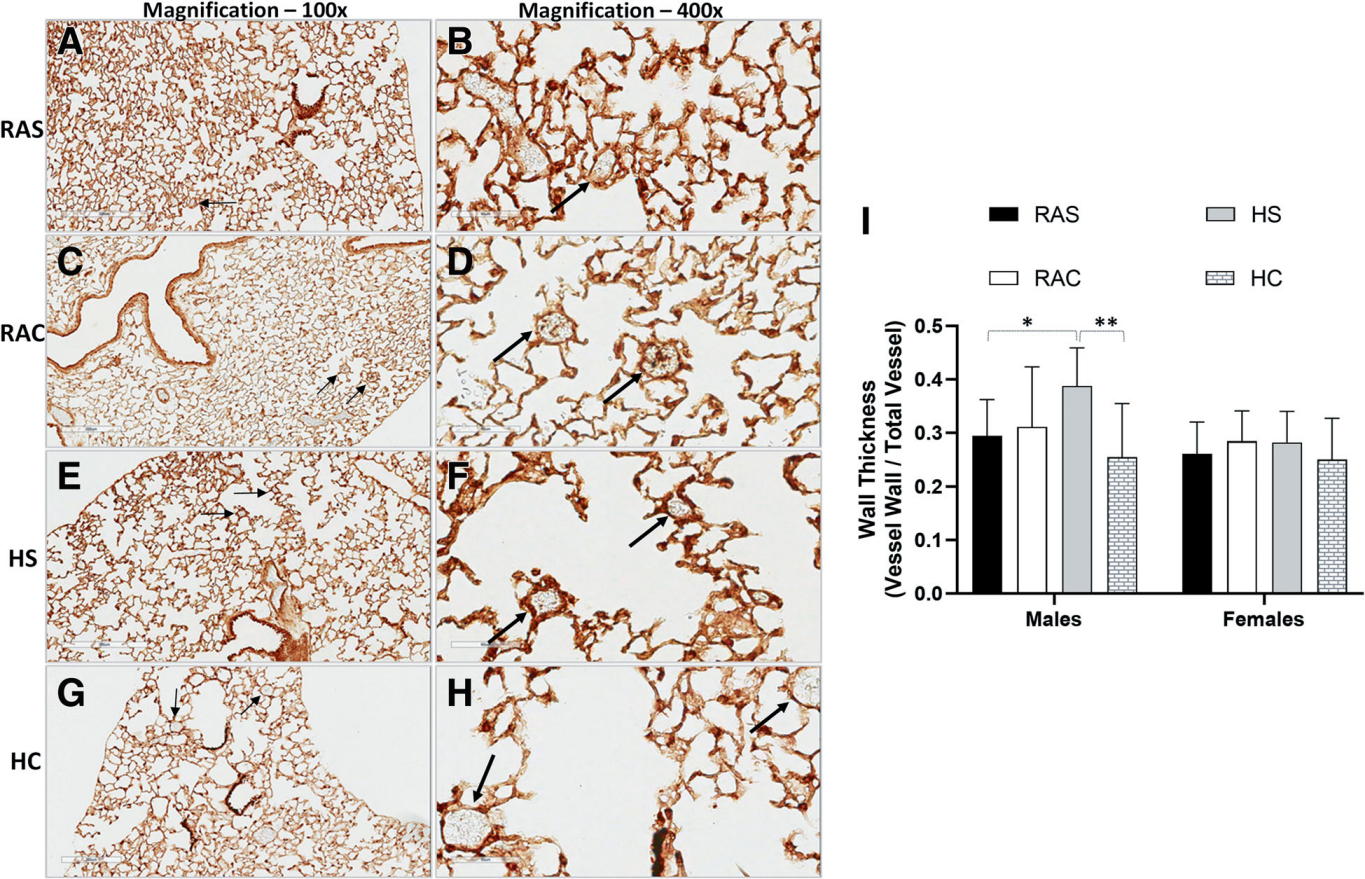
Assessment of vessel wall thickness at 12wks by α-smooth muscle actin immunohistochemistry of lung sections (100x: **a**/**c**/**e**/**g**; 400x: **b**/**d**/**f**/**h**; *room air saline (RAS):* A/B; *room air caffeine (RAC):* **c**/**d**; *hyperoxia saline (HS):* **e**/**f**; *hyperoxia caffeine (HC):* **g**/**h**). *Scale*: 200 μm (100x), 60 μm (400x). Thick arrows (400x) correspond to the same histologic location as thin arrows (100x) of the same group. Demonstrated vessels are of a 30-50 μm diameter in all the four groups. Pulmonary arterial vessel wall thickness was greater in the hyperoxia group suggestive of smooth muscle hypertrophy (**i**; *p < 0.001 vs. *RAS;* Fisher’s post-hoc test, ANOVA). Caffeine administration attenuated vessel wall thickness, especially in male mice at 12 weeks of age (**p < 0.0001 vs *HS* group, Fisher’s post-hoc test, ANOVA; **i**), suggesting it may have beneficial effects on the pulmonary vasculature (*Open box plots – RA groups; shaded box plots – hyperoxia groups*) (*n* = ten mice/group)

Assessment of pulmonary vascular smooth muscle thickness was performed in lung sections by α-SMA immunohistochemistry in all the groups (Fig. 4). There was no difference in the mean diameter of the pulmonary arterioles analyzed among the groups (*RAS:* 48.7 ± 4.0 μm; *RAC:* 50.2 ± 2.9 μm; *HS*: 48.4 ± 6.3 μm; *HC*: 51.9 ± 1.7 μm). Pulmonary arterioles were thicker with smooth muscle hypertrophy in mice exposed to neonatal hyperoxia group at 12wks (Fig. 4e/f). Postnatal caffeine in mice exposed to hyperoxia significantly reduced vessel wall thickness at 12wks, especially in male mice (*p < 0.001 vs. *RAS* & *RAC* groups; **p < 0.0001 vs. *HS* group; Fisher’s post-hoc test, ANOVA; Fig. 4i). Arterial wall thickness was not different among the groups in female mice. The effects of caffeine on pulmonary vascular remodeling and consequently on the development of pulmonary hypertension is gender specific with protective and beneficial effects in male mice.

## Discussion

Caffeine therapy for apnea of prematurity reduces the incidence of bronchopulmonary dysplasia in VLBW infants [5]. Although the mechanisms of the possible protective actions of caffeine are not apparent, potential effects on the endothelial cells [31, 32] and the vascular smooth muscle [33] in addition to the antagonism of the adenosine receptors [15] partly explain its beneficial effects. We have shown that caffeine by modulating the HIF-angiogenesis pathway, promotes alveolarization and angiogenesis with beneficial effects on the pulmonary vasculature.

**Fig. 5:**
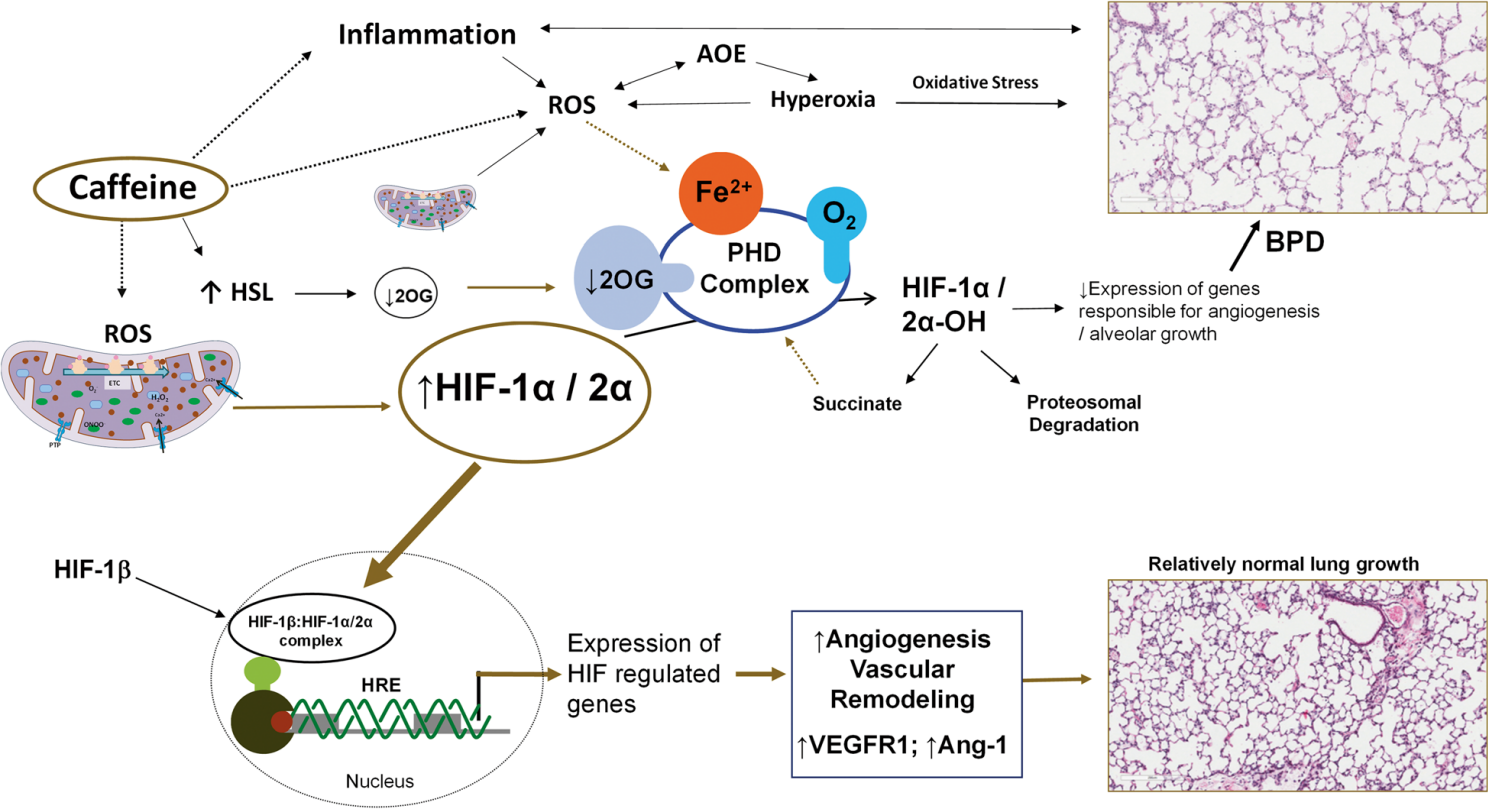
*Illustration of potential modulation of HIF regulation by caffeine*. Stabilization of HIF1α/2α determined by several factors including oxygen, the activity of PHD complex and its co-factors and reactive oxygen species. HIF-1α/2α activates multiple genes involved in glycolysis, angiogenesis, vascular remodeling, cell proliferation, and erythropoiesis via nuclear transcription. Higher expression of VEGFR1 and angiopoietin-1 along with vascular remodeling following caffeine administration may normalize lung histology seen in hyperoxia-induced lung injury. Hydroxylation of prolyl hydroxylase complex inactivates HIF-1α/2α followed by ubiquitination and proteasome degradation. HSL: hormone-sensitive lipase; 2OG: 2oxyglutarate; ROS: reactive oxygen species; PHD: prolyl hydroxylase; HIF: hypoxia inducible factor; HRE: hypoxia response element; VEGFR1: vascular endothelial growth factor receptor-1; Ang-1: angiopoietin-1; BPD: bronchopulmonary dysplasia; AOE – antioxidant enzyme activity; ETC – electron transport chain; PTP: permeability transition pore (*copyright – Vasantha HS Kumar, MD*)

Hypoxia-inducible factors (HIFs) are transcription factors that regulate the downstream expression of genes that mediate the response to hypoxia. Constant expression and degradation of HIF by PHD-2 results in not only a steady state concentration but also in a short half-life. In our study, both caffeine and hyperoxia by themselves significantly increased expression of HIF-2α and angiogenic genes VEGF and ANG-1. Figure 5 illustrates the potential role of caffeine in the modulation and regulation of HIF induced angiogenesis. Reactive oxygen species, including H_2_O_2,_ play a significant role in the upregulation of HIF pathway by several mechanisms, including interactions with PHD complex co-factors such as ferrous ion, ascorbate, and succinate [34]. Other intermediates such as nitric oxide, specific microRNAs and transcriptional and post-translational modifications mediate ROS mediated HIF-1 signaling [35]. Studies have shown a paradoxical higher level of HIF-1α in the presence of hyperoxia [36, 37]. Low concentrations of caffeine actively induce VEGF expression via activation of HIF-1α [38]. Transcript levels of HIF are of little predictive value concerning protein expression [39], as this can be influenced by protein half-life, post-translational changes, experimental conditions, and by the activity of prolyl hydroxylase isoforms. However, postnatal caffeine significantly induced HIF-2α protein in the lung in mice exposed to hyperoxia. HIF-2α could contribute to the expression of angiogenic genes seen in our study.

Angiogenic proteins, VEGF and its receptor, VEGFR1 was higher in both the hyperoxia groups; however, the receptor protein was significantly higher in the hyperoxia-exposed mice that received postnatal caffeine. While some animal studies have reported a relative decrease in lung VEGF mRNA and protein in response to hyperoxia [40–42], others have shown an increase in VEGF protein [43]. The variability in expression may be related to the stage of lung development in an animal model, the timing of the VEGF mRNA and its protein measurement and the variability of the VEGF isoforms measured [44]. The VEGF assay measured both 120 and 164 isoforms, and the expression of both proteins decreases with progression of lung development with low levels in adult lung. Mice in the hyperoxia-caffeine group had higher HIF-2α protein and VEGFR1 receptor density with relatively normal VEGF protein levels in the lung. Mice exposed to hyperoxia alone had significantly low levels of ANG1, a protein that plays a critical role in mediating reciprocal interactions between the endothelium, surrounding matrix, and the mesenchyme. ANG1 protein enhances the effect of VEGF, contributing to blood vessel maturation and vascular remodeling. Caffeine by increasing ANG1 in the lung may act synergistically with VEGF in ameliorating the abnormal vascular response during the early stages of lung injury. Caffeine by modulating the levels of VEGF and angiopoietin via the HIF pathway not only alters the abnormal vascular responses but may also enhance alveolar and microvascular development in the lung in the presence of hyperoxia.

Vessel density and vessel surface area were higher following postnatal caffeine in hyperoxia-exposed mice. An increase in vasculature may be secondary to higher expression of angiogenic proteins VEGFR1 and ANG1 in the HC group. An increase in vessel wall thickness following neonatal hyperoxia may suggest developing pulmonary hypertension in the context of hyperoxic lung injury [45]. However, postnatal caffeine attenuated lung injury with a significant decrease in vessel wall thickness following neonatal hyperoxia. The effects of caffeine on vascular remodeling is gender specific, with substantial benefits in male mice. However, female mice that received postnatal caffeine may have had minimal vascular remodeling compared to RA controls. Sex differences in systemic blood pressures are evident in young adults and continue through adulthood, with men more likely to be hypertensive than women regardless of race or ethnicity [46], and this may be related to the protective effects of sex hormones in females [46]. Induction of HIF-2α and ANG1 may play an essential role in microvascular stability and vascular remodeling. Caffeine-induced endothelial-dependent relaxation mediated by nitric oxide, endothelial-independent relaxation mediated by phosphodiesterase inhibition [47] and adenosine receptor blockade [15] among other smooth muscle effects [33] is additional contributors to the beneficial effects of caffeine on the vascular smooth muscle.

Improvements in lung morphometry measurements, including secondary septal length, indicate the protective effect of neonatal caffeine administration on postnatal lung growth in mice exposed to hyperoxia in both male and female mice. Caffeine may modulate the dynamic interrelationships between VEGF and ANG1, two critical regulators of pulmonary vascular development early in the evolution of lung injury, contributing to improved alveolarization in adult mice. However, the protective effects of caffeine on alveolar development are debatable with several studies suggesting an adverse effect on alveolar development [10, 14] and others signifying beneficial effects [12, 13, 48]. Even though inflammatory cytokines studied in the lung were unremarkable, caffeine may either worsen inflammation by increasing neutrophil chemoattractant, CXCL-1 [14] or may have a protective effect on lung inflammation [11, 13]. An increase in eNOS activity and tetrahydrobiopterin levels may contribute to improved alveolar structure and angiogenesis in immature lungs [48]. Improvements in lung structure relating to alveolar growth and attenuation of vascular remodeling may add to higher body weight, especially in adult male mice following hyperoxia. However, programming of offspring towards excess growth may occur from alterations in the hypothalamic-pituitary-adrenocortical axis [49] or changes in adenosine receptor expression [50] that play a key role in growth and development following prenatal caffeine [51].

Caffeine may affect multiple pathways and gene expression, and the limitation of the study is its focus on angiogenesis and lung development. Results of protein and mRNA expression of HIF-1 and angiogenic genes following hyperoxia may be difficult to extrapolate to lung growth at 12 weeks of age. Also, as lung growth and gene expression are developmentally regulated, assessment at transitional time points would have been useful. Beneficial effects of caffeine on the pulmonary vasculature could not be confirmed by either pulmonary hemodynamic studies or heart remodeling indices. Caffeine acts on tissues everywhere in the body by inhibiting the adenine receptors and increases the respiratory drive, respiratory rate, diaphragm function, and diuresis, among other effects [52, 53]. Postnatal caffeine treatment improves pulmonary function in mid-childhood [54]. We did not assess the impact of caffeine on lung function. Caffeine dose of 20 mg/kg in mice is equivalent to 2.4 mg/kg in humans [55]. The treatment administered is well below the recommended dose of 20 mg/kg loading dose followed by 5-10 mg/kg as a maintenance dose in neonates for apnea of prematurity [5]. Most of the animal studies with caffeine have used doses similar to ours for more extended periods (10–21 days). Caffeine for 4 days is the shortest duration of administration for assessment of lung histology and morphometry. Despite low dosage of caffeine, we demonstrate beneficial effects on angiogenesis, alveolarization, and vascular remodeling in adult mice. Even small doses of postnatal caffeine may have beneficial effects on lung growth and function over long-term with benefits apparent in male mice.

Caffeine is extensively used in premature neonates in the treatment of apnea of prematurity and to decrease the incidence of BPD. Although administered in large doses for long periods in newborns, the toxic effects of caffeine are infrequent. We have reported the beneficial effects of postnatal caffeine on lung morphometry and microvascular development with the potential to attenuate pulmonary vascular remodeling in male mice. Caffeine may modulate HIF-induced angiogenesis in mice exposed to hyperoxia, contributing to improved lung growth in adult mice.

## Data Availability

The datasets used and analyzed during the current study are available from the corresponding author on reasonable request.

## Abbreviations

ANG1: Angiopoietin-1
BPD: Bronchopulmonary dysplasia;
ELISA: Enzyme-linked immunosorbent assay
eNOS: Endothelial nitric oxide synthase
HIF: Hypoxia-inducible factor
HPF: High power field
IL6: Interleukin-6
INF-γ: interferon γ
PCR: Polymerase chain reaction
PHD-2: Prolyl hydroxylase-2
RA: Room air
RAC: Radial alveolar count
TGF-β: Transforming growth factor-β
TNF-α: Tumor necrosis factor α
VEGF: Vascular endothelial growth factor
VEGFR1/FLT1: Vascular endothelial growth factor receptor-1
VLBW: Very low birth weight
